# Supplementary material for: Epidemiological and molecular evidence of intrafamilial transmission through sexual and vertical routes in Bahia, the state with the highest prevalence of HTLV-1 in Brazil
Source: PLoS Negl Trop Dis. 2023 Sep 28;17(9):e0011005. doi: 10.1371/journal.pntd.0011005 (PMC10593241; doi:10.1371/journal.pntd.0011005)
Supplement: Attachment — Submitted filename: Reply Reviewers.docx [file pntd.0011005.s001.docx]

**Reviewer #1:**

***Query 1. Sequences are too short to be informative. Authors need to generate full LTR sequence, and it would even be better to have a complete genome or another gene (env?). They sequenced part of the LTR as a marker of transmission. The segment may be too short to be sufficiently informative.***

**Reply.** The reviewer raises an important point, and indeed, we recognize that it would have been ideal to generate complete LTR sequences, especially even the complete HTLV-1 genome. Our sequencing analysis was limited by the number of patients whose DNA was available, as well as the amount of DNA available from those who provided samples. Our attempts to obtain additional samples were unfortunately unsuccessful. Thus, our efforts to obtain the entire LTR by PCR resulted were limited to the segments included in the manuscript. However, since the HTLV-1 LTR is the most hypervariable region, these segments do contain sufficient information with which to identify polymorphic viral signatures. The fragments generated (480 base pairs on average, of 750 pb of the whole LTR sequence) were sufficient to distinguish inter- and intrafamilial genotypes (signatures). Overall, we identified seven genetic signatures in the LTR sequences from 20 members of eight families. Two of these signatures were identified in three families: *aattcgat* (families #152, #154, #158) and *gatttaac* (families #152, #154, #157). Signatures that were identical to the index case within the same family were considered as vertical or sexual transmission, depending on the degree of relatedness (wife and husband or mother and children). These signatures served as transmission markers to determine transmission routes in accordance with epidemiological and risk factors of family members. To address the reviewer’s concern, we have included the following paragraphs in the discussion section (lines 312-351):

“Combining epidemiological data, sexual behavior of family members, and LTR HTLV-1 signatures, evidence indicating the likely pathways of intrafamilial spread of HTLV-1 was obtained. The HTLV-1 LTR region was selected as a potential marker to evaluate routes of viral transmission, since this is the genetic region of the virus with greatest hypervariability (28). The fragments generated (480 base pairs on average, out of the 750 bp length of the entire LTR region) were sufficient to distinguish both inter- and intrafamilial signatures. These signatures served as genetic markers to infer transmission routes in accordance with the epidemiological and risk factors associated with different family members. For example, evidence of intrafamilial sexual transmission was found in families #155, #156, #157 and #159 (index case and husband from 4^th^ marriage). In these families, multiple risk factors for HTLV-1 infection were identified, such as multiple sexual partners, past history of STI, and two partners with an identical signature. On the contrary, vertical HTLV-1 transmission was the likely route of intrafamilial HTLV-1 spread in families #153, #154 and #158, as the same signature was detected in both mothers and children. Interestingly, in family #154, one of the daughters (from the mother’s third marriage) who presented the same signature as her mother had been breastfed for more than 6 months, had never had sexual intercourse, and had not received any blood transfusions. In contrast, the epidemiological and sexual history of another daughter (from her mother’s first marriage), who presented a different LTR HTLV -1 signature than her mother, precludes the possibility of vertical transmission. The latter daughter was the only infected child (out of 12) from this marriage. As her deceased partner was confirmed to be HTLV-1-positive, she was most likely infected sexually. In family #152, although both the mother and one of her daughters were infected, the family pedigree, risk factors associated with HTLV-1 infection, and LTR signatures did not provide any evidence of vertical transmission. The mother (*gatttaac*), who had received a blood transfusion before serologic HTLV-1/2 screening of blood donors became mandatory in Brazil (36), gave birth to five children before receiving the blood transfusion. The infected daughter had multiple sexual partners, never used a condom, and her HTLV-1-LTR signature (*aattcgat*) differed from that of her mother. Finally, in family #159, it was possible to sequence the HTLV-1 LTR region in family members across three generations, which resulted in evidence of both sexual transmission (mother and fourth husband with the same HTLV-1-LTR signature) and vertical transmission (a daughter from the mother’s third marriage and her children presented the same signature) (*gattcgac*). The results obtained herein support the role of using genetic signatures as markers for relevant modes of HTLV-1 transmission. It should be emphasized that as the HTLV-1 genome presents great stability as evidenced by a nucleotide exchange rate of about 1% every 1000 years (30,41), it therefore constitutes an excellent molecular marker for the evaluation of transmission route (30). Moreover, the rate of genetic variation observed within this same HTLV-1 aA subtype is less than 0.5% (42).”

*Query 2.* ***What is the nucleotide difference between sequences within and between different families?***

***Reply****.* Due to limitations the amplification of the LTR region during sequencing, it was not possible to obtain more robust genetic information that would offer insight into differences among the nucleotides observed in this region. However, the 8 base-pair signatures that were obtained does offer some insight into differences between the families with respect to transmission route. For example, of the three sequences identified in family 154, two sequences had the same pattern (*gatttaac*), while another sequence had a different sequence (*aattcgat*) at the same location in the genome. The following sentence has been included in the results section (lines 269-272):

" Overall, seven unique genetic signatures were identified in the LTR sequences obtained from 20 members of eight families. Two signatures were identified in three families: *aattcgat* (families #152, #154, #158) and *gatttaac* (families #152, #154, #157)..

***Query 3. How many mutations per transmission chain do they report?***

**Reply:** The information requested by the reviewer is certainly of interest; however, due to the aforementioned sequencing limitations, it is unfortunately impossible to investigate the progression of mutations throughout the transmission chain. As our research aimed to investigate the occurrence of both sexual and vertical familial transmission in the state of Bahia, by combining epidemiologic data, risk factors for HTLV-1 infection, sexual behavior, and LTR signatures, we were able to find 6 signatures in the context of sexual transmission, 4 associated with vertical transmission, and one arising from probable blood transfusion. It is important to note that none of the signatures identified were correlated with a particular route of transmission.

***Query 4 Authors should sequence the full ltr through 2 distinct PCRs and if possible, generate a full genome sequence to really trengthen their affirmation of intrafamilial transmission.***

**Reply**: We agree with the reviewer that it would have been ideal to generate complete LTR sequences, especially with respect to the complete HTLV-1 genome. Unfortunately, our sequencing analysis was limited by the number of patients whose DNA was available, as well as the amount of DNA available from those who provided samples; moreover, our attempts to obtain additional samples were ultimately unsuccessful. Thus, we are unable to offer additional insight beyond the genetic information in the LTR segments detailed in the manuscript. However, since the HTLV-1 LTR is the most hypervariable region, the segments obtained contain sufficient information for our purposes, which was to identify polymorphic viral signatures.

***Query 5 what is the supposed duration of breastfeeding? Authors report that most participants do not use condoms. Could they ask for the number of previous partners****.*
**Reply:** We have included a more detailed description of the epidemiological data, sexual behavior, and risk factors for HTLV-1 infection for those families whose DNA were sequenced, such as the occurrence of breastfeeding and duration, whether condoms were used, number of sexual partners, and history of blood transfusion.

To address the reviewer’s concern, we have included the following information in the Results section (Lines 195-269):

“In eight families, in addition to epidemiological data, it was possible to perform genetic sequencing of the LTR region to better assess the transmission routes of HTLV-1 (Figure 3). The index case of family #152 is a mother whose molecular LTR signature (*gatttaac*) differed from that of her daughter (*aattcgat*). She was diagnosed with HTLV-1 at age 70 during a routine examination. She reported being breastfed and had only two lifetime sexual partners (both with unknown HTLV serology). She received a blood transfusion in 1989, before HTLV-1 screening was mandatory for blood donors in Brazil (36). All five of her children, who were all born before she had received the blood transfusion, were breastfed for 6 months. Only one daughter was HTLV-1 positive by suspected sexual route of transmission. This infected daughter reported having five lifetime sexual partners and had never used a condom. Her two children were both HTLV-1 negative and were each breastfed for >6 months. The serological status of her children’s father is unknown. Family #153 is composed of a mother (index case) and one of her two HTLV-1-positive daughters with an identical LTR molecular signature (*gatacggc*), likely indicating infection by vertical route. The 84-year-old mother was diagnosed with HTLV-1 20 years ago, and has HAM/TSP. She became sexually activate at age 18, had 5 lifetime sexual partners and never used a condom. She has no reported history of blood transfusion. She had 8 children who were breastfed for > 6 months: three males (only one tested negative) and five females (three tested, two with positive serology). Vertical and sexual transmission likely both played a role in the familial clustering observed in family #154. The case index (the mother) exhibited an identical LTR signature (*gatttaac*) as one of her daughters, yet another daughter had a different signature (*aattcgat*). It is important to note that the mother was married three times. Of her 13 children from the first and second marriages, 12 were tested for HTLV and only one daughter (from the first marriage) was found to be infected. As this daughter had a different signature than her mother, she likely contracted the virus through sexual transmission, since she was married to an HTLV-1-positive partner (their daughter, who was breastfed for more than 6 months, also acquired the infection). The mother’s other HTLV-1-positive daughter from her third marriage was likely infected by vertical route, as she exhibited an identical LTR sequence. She was breastfed for more than 6 months, never had sexual intercourse, and never received a blood transfusion. At age 17, she was diagnosed with HAM/TSP. The likely cause of the HTLV-1 clustering observed in families #155, #156 and #157 is sexual transmission, as indicated by matching LTR signatures between index cases and their partners. In family #155 (150), the index case, the wife, was diagnosed with HAM/TSP in 2014 during routine serologic testing. She reported being breastfed, having only one sexual partner, and never using a condom. Additionally, she received a blood transfusion in 1987. Her husband had a history of multiple sexual partners (> 20) and STIs, was diagnosed with HTLV infection at the same time as his wife. The HTLV-1 serological status of the couple’s four children remains unknown. In family #156, the index case, the wife, was diagnosed with HTLV in 2012 when she presented neurological symptoms. She reported being breastfed, and both of her brothers tested negative for HTLV. Her first sexual intercourse occurred at the age of 15, and she had a total of 12 sexual partners without using condoms. She was married twice, had three children with unknown serology. Her second husband, diagnosed with HAM/TSP in 2016, had 15 sexual partners and no history of blood transfusion. In family #157, the index case (wife) was diagnosed with HTLV in the context of prenatal care in 2014, with HAM/TSP diagnosed in 2019. She does not have information about the serological status of her parents but reported being breastfed. She denies receiving blood transfusions. She reported having three sexual partners without using condoms. Despite her husband’s diagnosis of HTLV-1 in 2016, there is no available information on associated risk factors. The serologic status of their child remains unknown. In the case of family #158, the index case (daughter) was diagnosed in 2018 with HTLV-1 during prenatal testing. Her mother subsequently tested positive for HTLV, indicating a probable vertical transmission route. The index case had a normal delivery and did not breastfeed her baby. The 57-year-old mother has been married twice, but both of her husbands declined HTLV testing. She has a total of 13 children, all of whom were breastfed. Apart from the index case, two other daughters have also tested positive for HTLV-1. All of her sons refused to be tested. In Family #159, three generations of family members were evaluated. The index case of this family is a woman whose HTLV-1 LTR signature is identical to that of her two sons (*gattcgac*) and different from that of her mother (*gacttaac*), indicating a likely sexual route of HTLV-1 infection for the index case. She was breastfed, initiated sexual activity at the age of 14 and had six sexual partners, with the serology of five partners being unknown and the last partner having negative serology for HTLV. She received a diagnosis of HTLV-1 in 2013 when she exhibited symptoms of myelopathy. In 2018, she was diagnosed with HAM/TSP and died in 2020. Of her eight children with her last partner, seven underwent serology testing, and one refused testing. Of the six children who tested positive for HTLV-1, two underwent genetic sequencing, indicating vertical transmission as evidenced by identical LTR signatures to their mother. Two children experienced severe disseminated strongyloidiasis (37), and one of the two later died. Importantly, the last-born child with negative serology was not breastfed because the mother already knew she was infected. The mother of the index case was diagnosed with HTLV-1 in 2013 when she was already showing signs of myelopathy. She had been married four times. In the mother’s fourth marriage, both she and her HTLV-1-positive husband underwent genetic sequencing, with identical LTR signature (*gacttaac*). Both of them were diagnosed with syphilis."

***Query 6 Authors should discuss their data according to other publications of intrafamilial transmission: -    Other papers have looked at intrafamilial transmission and should be discussed and presented: Tuppin et al 1996 ; VanDooren et al, 2007***

**Reply:** We thank the reviewer for suggesting these articles by Tuppin et al 1996 and VanDooren et al 2007, which we have analyzed in the light of intrafamilial HTLV-1 transmission. The article by Tuppin P et al., 1996 details a cluster of pregnant women with indeterminate Western blot results for HTLV, which is unfortunately outside the scope of our study. (Tuppin P, Makuwa M, Guerma T, Bazabana MM, Loukaka JC, Jeannel D, M'Pelé P, de Thé G. Low HTLV-I/ II seroprevalence in pregnant women in Congo and a geographic cluster of an HTLV-like indeterminate western blot pattern. J Acquir Immune Defic Syndr Hum Retrovirol. 1996 Jan 1;11(1):105-7. doi: 10.1097/00042560-199601010-00014). We believe the second article suggested by the reviewer evaluated risk factors associated with HTLV-1 transmission as reported by Gotuzzo et al. 2007, which includes Van Dooren S as an author (Gotuzzo E, Moody J, Verdonck K, Cabada MM, González E, Van Dooren S, et al. Frequent HTLV-1 infection in the offspring of Peruvian women with HTLV-1–associated myelopathy/tropical spastic paraparesis or strongyloidiasis. Rev Panam Salud Publica. 2007;22(4):223–30.). We have included a sentence in the discussion section to call attention to the importance of evaluating concomitant diseases, such as strongyloidiasis (Lines 386-388).

“Moreover, the concomitant presence of HAM/TSP and strongyloidiasis in HTLV-1-infected mothers was reported to be associated with higher seropositivity in children (51).

**Reviewer #2:**

***Query 1- The aims are stated in the Introduction (lines 94-96): "to investigate familial clustering of HTLV-1 infection in the state of Bahia, as well as to determine likely routes of transmission through epidemiological and genetic analysis". Please could the authors provide more information on the rationale for the use of viral genetic analysis to investigate familial clustering of HTLV-1, including defining the term 'intrafamilial polymorphisms' and how these could provide evidence of vertical versus sexual transmission.***

**Reply***.* We have included a paragraph in the introduction section explaining the rationale for using genetic analysis as a tool to evaluate intrafamilial transmission routes (lines 95-101).

“The performance of viral genetic analysis to identify intrafamilial polymorphisms, i.e., variations in the genetic sequence of a virus within a particular family, can provide insight into the mode of HTLV-1 transmission. While the evolutionary rate of HTLV-1 is quite low, the long terminal repeat (LTR) and envelope (env) regions of the virus exhibit higher variability compared with other genomic regions (28,29). This variability serves as a relevant molecular marker for monitoring transmission pathways and can be used to distinguish between sexual and vertical transmission”.

***Query 2. Please could some further details on the population be added. In particular, were family members tested through CHTLV or other clinics? Do we know how many family members were offered a test and declined?***

**Reply**. Unfortunately, we do not have precise information regarding the number of family members who consented to or declined HTLV-1 testing. Out of the 1,759 patients from CHTLV who were assessed to identify whether their first-degree relatives were tested, at least one family member underwent testing in 424 (24.1%) cases. It is crucial to emphasize that CHTLV recommends and offers HTLV-1 testing to all family members of enrolled patients. However, high refusal rates are significant concern, and we believe that this plays an important role in the silent spread of HTLV-1 within families. To highlight this concern, we have included a statement to call attention to this issue (Lines 374-382).

" In addition, the silent dissemination of HTLV reflects a complex network of factors that impose limitations on access to testing, such as a lack of knowledge about HTLV among health professionals, deficient testing availability, few campaigns specifically aimed at preventing sexual transmission, and patient/family fear of social stigma. On the other hand, the introduction of public policies can interrupt the chain of HTLV-1 transmission if infected women are encouraged to stop breastfeeding and receive infant formula for their children, as observed in families #158 and #159. In addition, it was recently reported that mandatory HTLV screening and the cessation of breastfeeding are cost-effective measures in Brazil (50)”.

***Query 3. Are there concerns about ethical or regulatory requirements being met?
The authors mention (line 268) that written informed consent was not required according to national and local specifications. Was oral consent required and if so, obtained, eg for collection of blood samples for HTLV sequencing, or completion of semi-structured questionnaires?***

**Reply**. The study was approved by the Institutional Research Board of EBMSP (CAAE: 60554416.8.0000.5544), and informed consent was obtained whenever possible. In accordance with the ethical principles outlined in Brazilian regulations, the need for consent was waived only in the case of death or loss to follow-up.

We have included this information in the manuscript.

***Query 4. Spouses were identified, who had been tested for HTLV, but not unmarried partners. Could the latter also be included?***

**Reply**. We appreciate the reviewer’s concern and have included a more detailed description of all family members, whether married or not, regardless of HTLV test results. We have additionally included the number of sexual partners of the HTLV-infected individuals. However, all of the information regarding each individual’s HTLV infection status has been provided in our results (Lines 195-269):

***Query 5. Is the proportion of medical records lacking adequate documentation on first-degree relative HTLV testing available?***

**Reply.** We have revised the pedigrees shown in Figures 2 and 3, using all available medical records from the index cases with respect to HTLV testing of family members. Unfortunately, in most cases, several first-degree members were not tested. The updated figures indicate whether the test results available were positive, negative, or unknown.

***Query 6. Why did the authors exclude patients and family members with cognitive impairment, psychiatric/psychological disorders and those with HIV/HBV/HCV? The rationale should be stated. For example, many individuals with HTLV-1 may experience psychological disorders such as depression and excluding them may introduce bias.***

**Reply:** Individuals with marked cognitive deficits or relevant psychiatric disorders may not be able to adequately respond to the items on the questionnaire used. In addition, we excluded patients with other coinfections (HIV, HCV, and HBV) because familial clustering has already been reported for some of these diseases, which could lead to overestimation. In the present study, five patients (index cases) were excluded, all due to HTLV-2 (2), HCV (1) and cognitive impairment (2). This information is now detailed Figure 1.

***Query 7. Questionnaires: were these completed by patients or healthcare professionals? If the latter, please state the sources used to complete them.***

**Reply**: Questionnaires were completed by a single researcher (ANS) during a nursing appointment at the center. Additional information was obtained from patients' medical records.

***Query 8. Genetic sequencing: why were only a subset of individuals included? Were these fresh or stored samples? Presumably they were whole blood specimens, which should be stated. Please also state the rationale for choosing LTR rather than another viral gene region or whole genome sequencing.***

**Reply**: We performed genetic sequencing only on patients whose DNA samples were available in our repository of samples. We included the following paragraph in the Methods section (127-128) Lines (127-128):

“DNA was extracted using a QIAamp DNA Blood Mini Kit (Qiagen, Hilden, Germany) in accordance with the manufacturer's instructions, and then stored at -20^o^C until use.”

The reviewer raises an important point. Indeed, it would be more informative to have the complete LTR sequence or even the complete HTLV-1 genome. However, the amount of DNA available was not sufficient, so it was impossible to amplify a larger fragment. Therefore, we decided to amplify the LTR region of HTLV-1, which is the most hypervariable region. The fragments generated (on average 480 base pairs out of 750 pb of the total LTR sequence) were sufficient to distinguish between inter- and intrafamilial genotypes (signatures). We have included a paragraph in the Discussion to clarify this issue (Lines 312-351).

“Combining epidemiological data, sexual behavior of family members, and LTR HTLV-1 signatures, evidence indicating the likely pathways of intrafamilial spread of HTLV-1 was obtained. The HTLV-1 LTR region was selected as a potential marker to evaluate routes of viral transmission, since this is the genetic region of the virus with greatest hypervariability (28). The fragments generated (480 base pairs on average, out of the 750 bp length of the entire LTR region) were sufficient to distinguish both inter- and intrafamilial signatures. These signatures served as genetic markers to infer transmission routes in accordance with the epidemiological and risk factors associated with different family members. For example, evidence of intrafamilial sexual transmission was found in families #155, #156, #157 and #159 (index case and husband from 4^th^ marriage). In these families, multiple risk factors for HTLV-1 infection were identified, such as multiple sexual partners, past history of STI, and two partners with an identical signature. On the contrary, vertical HTLV-1 transmission was the likely route of intrafamilial HTLV-1 spread in families #153, #154 and #158, as the same signature was detected in both mothers and children. Interestingly, in family #154, one of the daughters (from the mother’s third marriage) who presented the same signature as her mother had been breastfed for more than 6 months, had never had sexual intercourse, and had not received any blood transfusions. In contrast, the epidemiological and sexual history of another daughter (from her mother’s first marriage), who presented a different LTR HTLV -1 signature than her mother, precludes the possibility of vertical transmission. The latter daughter was the only infected child (out of 12) from this marriage. As her deceased partner was confirmed to be HTLV-1-positive, she was most likely infected sexually. In family #152, although both the mother and one of her daughters were infected, the family pedigree, risk factors associated with HTLV-1 infection, and LTR signatures did not provide any evidence of vertical transmission. The mother (*gatttaac*), who had received a blood transfusion before serologic HTLV-1/2 screening of blood donors became mandatory in Brazil (36), gave birth to five children before receiving the blood transfusion. The infected daughter had multiple sexual partners, never used a condom, and her HTLV-1-LTR signature (*aattcgat*) differed from that of her mother. Finally, in family #159, it was possible to sequence the HTLV-1 LTR region in family members across three generations, which resulted in evidence of both sexual transmission (mother and fourth husband with the same HTLV-1-LTR signature) and vertical transmission (a daughter from the mother’s third marriage and her children presented the same signature) (*gattcgac*). The results obtained herein support the role of using genetic signatures as markers for relevant modes of HTLV-1 transmission. It should be emphasized that as the HTLV-1 genome presents great stability as evidenced by a nucleotide exchange rate of about 1% every 1000 years (30,41), it therefore constitutes an excellent molecular marker for the evaluation of transmission route (30). Moreover, the rate of genetic variation observed within this same HTLV-1 aA subtype is less than 0.5% (42).”

***Query 9. The genetic results need further explanation within the text ie how they were interpreted to assign sexual or vertical transmission routes.*
Reply***. To clarify this point, we have included the following paragraph in the Methods section (Lines 141-150):*

**“Evaluation of HTLV-1 familial aggregation**

Familial aggregation was considered when more than one member of the same family tested positive for HTLV-1, with the index case being the first family member registered at CHTLV. Semi-structured questionnaires were used to collect sociodemographic, clinical, and behavioral data. Patients with HAM/TSP were diagnosed according to previously described criteria (35). To determine the likely route of HTLV-1 infection, epidemiologic data and risk factors from family members were evaluated in combination with available LTR sequence signatures. Identical signatures between index cases in the same family were considered as evidence of vertical or sexual transmission, depending on the degree of relatedness (mother and siblings, or wife and husband). “

***Query 10 Figure 1 - please add % as well as total numbers. The authors should later discuss why the family members of most patients (1335/1759) had not been tested for HTLV.***

**Reply**: We have modified the Figure 1 and included the percentages. We also included a paragraph in the discussion section to explain why so many family members has not been tested for HTLV (Lines 374-378):

“In addition, the silent dissemination of HTLV reflects a complex network of factors that impose limitations on access to testing, such as: a lack of knowledge about HTLV among health professionals, deficient testing availability, few campaigns specifically aimed at preventing sexual transmission, and patient/family fear of social stigma.”

***Query 11. Figure 3 - show transmission route categories of vertical / sexual / both routes for the different families***

**Reply***:* We have modified the Figure 3 as requested.

***Query 12. Table 1 - for widowed patients, are the deaths of any partners considered likely to be HTLV related?***

**Reply**: We only considered HTLV-1 as a cause of death when the infection was documented

***Query 13. Table 2 - add ATLL diagnoses***

**Reply**: We do not have information about patients with ATLL diagnosis.

***Query 14. General - are proviral load data available? It would be interesting to note proviral load in patients with and without HTLV-1 positive first-degree relatives, for example.*
Reply:** Data on HTLV-1 proviral load are available for some patients, and an evaluation in the groups of patients with and without HTLV-1-positive first-degree relatives would be interesting. However, this was not the aim of this study.

***Query 15. General - the authors mention reporting of condom use, breastfeeding and*** HTLV-1 LTR was performed for some family members for whom DNA samples were available ***receipt of blood transfusions. Are any of the following data also available? Duration of breastfeeding, history of injection drug use, history of organ transplant***

**Reply**: We have included a more detailed description of patients for whom DNA samples were available on results section (Lines 195-274)

‘In eight families, in addition to epidemiological data, it was possible to perform genetic sequencing of the LTR region to better assess the transmission routes of HTLV-1 (Figure 3). The index case of family #152 is a mother whose molecular LTR signature (*gatttaac*) differed from that of her daughter (*aattcgat*). She was diagnosed with HTLV-1 at age 70 during a routine examination. She reported being breastfed and had only two lifetime sexual partners (both with unknown HTLV serology). She received a blood transfusion in 1989, before HTLV-1 screening was mandatory for blood donors in Brazil (36). All five of her children, who were all born before she had received the blood transfusion, were breastfed for 6 months. Only one daughter was HTLV-1 positive by suspected sexual route of transmission. This infected daughter reported having five lifetime sexual partners and had never used a condom. Her two children were both HTLV-1 negative and were each breastfed for >6 months. The serological status of her children’s father is unknown. Family #153 is composed of a mother (index case) and one of her two HTLV-1-positive daughters with an identical LTR molecular signature (*gatacggc*), likely indicating infection by vertical route. The 84-year-old mother was diagnosed with HTLV-1 20 years ago, and has HAM/TSP. She became sexually activate at age 18, had 5 lifetime sexual partners and never used a condom. She has no reported history of blood transfusion. She had 8 children who were breastfed for > 6 months: three males (only one tested negative) and five females (three tested, two with positive serology). Vertical and sexual transmission likely both played a role in the familial clustering observed in family #154. The case index (the mother) exhibited an identical LTR signature (*gatttaac*) as one of her daughters, yet another daughter had a different signature (*aattcgat*). It is important to note that the mother was married three times. Of her 13 children from the first and second marriages, 12 were tested for HTLV and only one daughter (from the first marriage) was found to be infected. As this daughter had a different signature than her mother, she likely contracted the virus through sexual transmission, since she was married to an HTLV-1-positive partner (their daughter, who was breastfed for more than 6 months, also acquired the infection). The mother’s other HTLV-1-positive daughter from her third marriage was likely infected by vertical route, as she exhibited an identical LTR sequence. She was breastfed for more than 6 months, never had sexual intercourse, and never received a blood transfusion. At age 17, she was diagnosed with HAM/TSP. The likely cause of the HTLV-1 clustering observed in families #155, #156 and #157 is sexual transmission, as indicated by matching LTR signatures between index cases and their partners. In family #155 (150), the index case, the wife, was diagnosed with HAM/TSP in 2014 during routine serologic testing. She reported being breastfed, having only one sexual partner, and never using a condom. Additionally, she received a blood transfusion in 1987. Her husband had a history of multiple sexual partners (> 20) and STIs, was diagnosed with HTLV infection at the same time as his wife. The HTLV-1 serological status of the couple’s four children remains unknown. In family #156, the index case, the wife, was diagnosed with HTLV in 2012 when she presented neurological symptoms. She reported being breastfed, and both of her brothers tested negative for HTLV. Her first sexual intercourse occurred at the age of 15, and she had a total of 12 sexual partners without using condoms. She was married twice, had three children with unknown serology. Her second husband, diagnosed with HAM/TSP in 2016, had 15 sexual partners and no history of blood transfusion. In family #157, the index case (wife) was diagnosed with HTLV in the context of prenatal care in 2014, with HAM/TSP diagnosed in 2019. She does not have information about the serological status of her parents but reported being breastfed. She denies receiving blood transfusions. She reported having three sexual partners without using condoms. Despite her husband’s diagnosis of HTLV-1 in 2016, there is no available information on associated risk factors. The serologic status of their child remains unknown. In the case of family #158, the index case (daughter) was diagnosed in 2018 with HTLV-1 during prenatal testing. Her mother subsequently tested positive for HTLV, indicating a probable vertical transmission route. The index case had a normal delivery and did not breastfeed her baby. The 57-year-old mother has been married twice, but both of her husbands declined HTLV testing. She has a total of 13 children, all of whom were breastfed. Apart from the index case, two other daughters have also tested positive for HTLV-1. All of her sons refused to be tested. In Family #159, three generations of family members were evaluated. The index case of this family is a woman whose HTLV-1 LTR signature is identical to that of her two sons (*gattcgac*) and different from that of her mother (*gacttaac*), indicating a likely sexual route of HTLV-1 infection for the index case. She was breastfed, initiated sexual activity at the age of 14 and had six sexual partners, with the serology of five partners being unknown and the last partner having negative serology for HTLV. She received a diagnosis of HTLV-1 in 2013 when she exhibited symptoms of myelopathy. In 2018, she was diagnosed with HAM/TSP and died in 2020. Of her eight children with her last partner, seven underwent serology testing, and one refused testing. Of the six children who tested positive for HTLV-1, two underwent genetic sequencing, indicating vertical transmission as evidenced by identical LTR signatures to their mother. Two children experienced severe disseminated strongyloidiasis (37), and one of the two later died. Importantly, the last-born child with negative serology was not breastfed because the mother already knew she was infected. The mother of the index case was diagnosed with HTLV-1 in 2013 when she was already showing signs of myelopathy. She had been married four times. In the mother’s fourth marriage, both she and her HTLV-1-positive husband underwent genetic sequencing, with identical LTR signature (*gacttaac*). Both of them were diagnosed with syphilis. Overall, seven unique genetic signatures were identified in the LTR sequences obtained from 20 members of eight families. Two signatures were identified in three families: *aattcgat* (families #152, #154, #158) and *gatttaac* (families #152, #154, #157). Of note, the cosmopolitan HTLV-1 subtype of transcontinental subgroup A was identified by sequencing the HTLV-1 LTR region in the samples examined from all families.”

***Query 16 Further details should be added eg speculating on why the % of tested family members was low, and how this could be improved. Also, any limitations around how they concluded vertical versus sexual transmissions within families.***

**Reply***:* In fact, the number of family members that refuse to be tested for HTLV-1 infection or the number of untested members in a family with an identified case of infection is high. We consider that is a complex problem, due to multiple factors. We have included the following paragraph in the discussion (Lines 374-378):

“In addition, the silent dissemination of HTLV reflects a complex network of factors that impose limitations on access to testing, such as: a lack of knowledge about HTLV among health professionals, deficient testing availability, few campaigns specifically aimed at preventing sexual transmission, and patient/family fear of social stigma.”

***Query 17. There is some discussion, but this could be improved, for example, considering current barriers to family member testing; discussing in more detail the interventions which may reduce sexual transmission of HTLV-1 (clinician/patient awareness, increasing testing in STI clinics, promotion of condom use etc)***

**Reply:** We have included the following paragraph in the discussion section (lines 367-382):

“The silent intrafamilial spread of infection via multiple modes of transmission within the same family over several generations highlights the neglected status of HTLV-1, as well as the social vulnerability associated with this infection. For example, family #159 lives in extreme poverty in a semi-isolated community with poor sanitary conditions(49). The index case (diagnosed with HAM/TSP) died at the age of 42 years, had six children infected with HTLV-1, one of whom developed disseminated strongyloidiasis and later died (37). Cases of Norwegian scabies have also been observed in this family (unpublished data). In addition, the silent dissemination of HTLV reflects a complex network of factors that impose limitations on access to testing, such as a lack of knowledge about HTLV among health professionals, deficient testing availability, few campaigns specifically aimed at preventing sexual transmission, and patient/family fear of social stigma. On the other hand, the introduction of public policies can interrupt the chain of HTLV-1 transmission if infected women are encouraged to stop breastfeeding and receive infant formula for their children, as observed in families #158 and #159. In addition, it was recently reported that mandatory HTLV screening and the cessation of breastfeeding are cost-effective measures in Brazil (50)”.

***Query 18. Line 218 - clarify which measures in Japan were implemented.***

**Reply***:* We have included the following the information as requested (Lines 353-356): “Measures implemented in Japan to control vertical HTLV-1 transmission, such as screening pregnant women in endemic areas and formula-feeding the babies of infected women, have successfully resulted in significantly decreased prevalence (43).”

***Query 19. Line 221 - since the introduction of mandatory HTLV testing for pregnant women in Bahia, what has been the uptake and % positivity?***

**Reply***.* This is an interesting question; we are currently performing a study to address this question.

**Query 20. C**larifications are required on the background and rationale for the use of HTLV-1 genetic analyses, and how these results were interpreted.

**Reply**: We have included the following paragraph in the introduction section (lines 95-101):

“The performance of viral genetic analysis to identify intrafamilial polymorphisms, i.e., variations in the genetic sequence of a virus within a particular family, can provide insight into the mode of HTLV-1 transmission. While the evolutionary rate of HTLV-1 is quite low, the long terminal repeat (LTR) and envelope (env) regions of the virus exhibit higher variability compared with other genomic regions (28,29). This variability serves as a relevant molecular marker for monitoring transmission pathways and can be used to distinguish between sexual and vertical transmission (25,30,31)”.

We also have included in the discussion details of how we interpretate the results of the genotype analysis, by combing epidemiological, sexual behavior and risk factors to HTLV-1 infection. We hope we made clear that HTLV-1 signatures are an additional element to determine the route of HTLV-1 infection (Lines 312- 351).

“Combining epidemiological data, sexual behavior of family members, and LTR HTLV-1 signatures, evidence indicating the likely pathways of intrafamilial spread of HTLV-1 was obtained. The HTLV-1 LTR region was selected as a potential marker to evaluate routes of viral transmission, since this is the genetic region of the virus with greatest hypervariability (28). The fragments generated (480 base pairs on average, out of the 750 bp length of the entire LTR region) were sufficient to distinguish both inter- and intrafamilial signatures. These signatures served as genetic markers to infer transmission routes in accordance with the epidemiological and risk factors associated with different family members. For example, evidence of intrafamilial sexual transmission was found in families #155, #156, #157 and #159 (index case and husband from 4^th^ marriage). In these families, multiple risk factors for HTLV-1 infection were identified, such as multiple sexual partners, past history of STI, and two partners with an identical signature. On the contrary, vertical HTLV-1 transmission was the likely route of intrafamilial HTLV-1 spread in families #153, #154 and #158, as the same signature was detected in both mothers and children. Interestingly, in family #154, one of the daughters (from the mother’s third marriage) who presented the same signature as her mother had been breastfed for more than 6 months, had never had sexual intercourse, and had not received any blood transfusions. In contrast, the epidemiological and sexual history of another daughter (from her mother’s first marriage), who presented a different LTR HTLV -1 signature than her mother, precludes the possibility of vertical transmission. The latter daughter was the only infected child (out of 12) from this marriage. As her deceased partner was confirmed to be HTLV-1-positive, she was most likely infected sexually. In family #152, although both the mother and one of her daughters were infected, the family pedigree, risk factors associated with HTLV-1 infection, and LTR signatures did not provide any evidence of vertical transmission. The mother (*gatttaac*), who had received a blood transfusion before serologic HTLV-1/2 screening of blood donors became mandatory in Brazil (36), gave birth to five children before receiving the blood transfusion. The infected daughter had multiple sexual partners, never used a condom, and her HTLV-1-LTR signature (*aattcgat*) differed from that of her mother. Finally, in family #159, it was possible to sequence the HTLV-1 LTR region in family members across three generations, which resulted in evidence of both sexual transmission (mother and fourth husband with the same HTLV-1-LTR signature) and vertical transmission (a daughter from the mother’s third marriage and her children presented the same signature) (*gattcgac*). The results obtained herein support the role of using genetic signatures as markers for relevant modes of HTLV-1 transmission. It should be emphasized that as the HTLV-1 genome presents great stability as evidenced by a nucleotide exchange rate of about 1% every 1000 years (30,41), it therefore constitutes an excellent molecular marker for the evaluation of transmission route (30). Moreover, the rate of genetic variation observed within this same HTLV-1 aA subtype is less than 0.5% (42).”
