# Supplementary material for: Epidemiological and molecular evidence of intrafamilial transmission through sexual and vertical routes in Bahia, the state with the highest prevalence of HTLV-1 in Brazil
Source: PLoS Negl Trop Dis. 2023 Sep 28;17(9):e0011005. doi: 10.1371/journal.pntd.0011005 (PMC10593241; doi:10.1371/journal.pntd.0011005)
Supplement: Attachment — Submitted filename: Reply Reviewer V2.docx [file pntd.0011005.s002.docx]

Query Reviewer #1: I would just ask the authors to add in the discussion a remark they made to my comments: "It is important to note that none of the signatures identified were correlated with a particular route of transmission."

**Reply:** We have included in the discussion section the remark as requested (lines 346-347)
